# Bioenergetic variation is related to autism symptomatology

**DOI:** 10.1007/s11011-017-0087-0

**Published:** 2017-08-29

**Authors:** Leanna Delhey, Ekim Nur Kilinc, Li Yin, John Slattery, Marie Tippett, Rebecca Wynne, Shannon Rose, Stephen Kahler, Shirish Damle, Agustin Legido, Michael J. Goldenthal, Richard E. Frye

**Affiliations:** 1 Autism Research Program, Arkansas Children’s Research Institute, Slot 512-41B, 13 Children’s Way, Little Rock, AR 72202 USA; 2 Department of Pediatrics, University of Arkansas for Medical Sciences, Little Rock, AR 72202 USA; 3 West China Hospital of Sichuan University, Nanchong, Sichuan China; 4 Department of Pediatrics, Drexel University College of Medicine Neurology Section, St. Christopher’s Hospital for Children, Philadelphia, PA 19134 USA

**Keywords:** Autism Spectrum disorder, Complex I, Complex IV, Electron transport chain, Mitochondrial dysfunction

## Abstract

Autism spectrum disorder (ASD) has been associated with mitochondrial dysfunction but few studies have examined the relationship between mitochondrial function and ASD symptoms. We measured Complex I and IV and citrate synthase activities in 76 children with ASD who were not receiving vitamin supplementation or medication. We also measured language using the Preschool Language Scales or Clinical Evaluation of Language Fundamentals, adaptive behavior using the Vineland Adaptive Behavioral Scale, social function using the Social Responsiveness Scale and behavior using Aberrant Behavior Checklist, Childhood Behavior Checklist and the Ohio Autism Clinical Impression Scale. Children with ASD demonstrated significantly greater variation in mitochondrial activity compared to controls with more than expected ASD children having enzyme activity outside of the normal range for Citrate Synthase (24%), Complex I (39%) and Complex IV (11%). Poorer adaptive skills were associated with Complex IV activity lower or higher than average and lower Complex I activity. Poorer social function and behavior was associated with relatively higher Citrate Synthase activity. Similar to previous studies we find both mitochondrial underactivity and overactivity in ASD. This study confirms an expanded variation in mitochondrial activity in ASD and demonstrates, for the first time, that such variations are related to ASD symptoms.

## Background

Autism spectrum disorder (ASD) is a behaviorally defined disorder which has continued to rise in prevalence over the last two decades and now affects ~2% of children in the United States (Zablotsky et al. 2015). Although its etiology remains poorly understood, recent studies suggest a link to mitochondrial dysfunction (Frye and Rossignol 2011; Rossignol and Frye 2012; Legido et al. 2013). The exact nature of mitochondrial dysfunction and its link to symptoms, however, remains an area of active investigation. A recent meta-analysis demonstrated that classic mitochondrial disease is found in approximately 5% (Rossignol and Frye 2012) of children with ASD, yet 30–50% have biomarkers of mitochondrial dysfunction (Frye 2012a, b; Rossignol and Frye 2012) and a higher rate of abnormal electron transport chain (ETC) activity has been documented in lymphocytes and granulocytes (Giulivi et al. 2010; Napoli et al. 2014) and post-mortem brain tissue (Palmieri et al. 2010). This suggests that ASD may have a unique relationship to abnormal mitochondrial function.

Major criteria that define classic mitochondrial disease include unequivocal genetic mutations, severe depressions (i.e., <30%) in ETC function or syndromic presentation (Frye and Rossignol 2011). However, our meta-analysis demonstrated that the minority (~25%) of children with ASD and mitochondrial disease have identifiable genetic mutations that explain the mitochondrial disease (Rossignol and Frye 2012) and many case reports and series have described moderate, rather than severe, deficiencies in ETC activity (Frye 2012a, b, 2013b). Perhaps more unique is the fact that ETC activity in muscle (Frye and Naviaux 2011; Frye 2012a, b), skin (Frye et al. 2013b), buccal epithelium(Legido et al. 2013; Goldenthal et al. 2015; Frye et al. 2016a) and brain (Palmieri et al. 2010) has been documented to be significantly increased, rather than decreased, in individuals with ASD. This is consistent with our in vitro data showing elevated mitochondrial respiration in lymphoblastoid cell lines (LCLs) derived from children with ASD (Rose et al. 2014a, b; Frye et al. 2016c; Rose et al. 2017).

In order to investigate variation in ETC function in children with ASD without using invasive procedures like a muscle biopsy, Goldenthal et al. (Goldenthal et al. 2015) used a validated buccal swab method (Goldenthal et al. 2012) to collect data on the largest sample to date. Goldenthal et al. (Goldenthal et al. 2015) verified the expanded variation in mitochondrial function in children with ASD and demonstrated that children with ASD demonstrated both depressions and elevations in ETC function. Still the relationship between ETC complex activity and cognitive and behavioral symptoms has not been investigated in a large sample. Thus this study uses the buccal swab technique to examine ETC function in a large sample of ASD children and determine the correspondence between variations in mitochondrial function and cognitive development and behavior. Most importantly, participants in this study were not taking any supplements or medications that could interfere with mitochondrial function at the time of testing.

## Methods

The study was approved by the Institutional Review Board at the University of Arkansas for Medical Sciences (Little Rock, AR). Parents of participants provided written informed consent. All experiments were performed in accordance with relevant guidelines and regulations.

### ASD participants

76 individuals with ASD who met inclusion and exclusion criteria had ETC complex function measured with 61 undergoing cognitive and behavioral evaluations. Clinical characteristics of the participants are provided in Table 1.

**Table 1:** ASD participant characteristics (*N* = 76)

Variable	
Age, mean (SD), years months	8 years 9 months (3 years 11 months)
Males, N (%)	55 (72.4%)
Language Testing, N (%)	
Preschool Language Scales	19 (25.0%)
Clinical Evaluation of Language Fundamentals 2	4 (5.3%)
Clinical Evaluation of Language Fundamentals 4	38 (50%)
ASD Diagnostic Documentation, N (%)	
Autism Diagnostic Observation Schedule	16 (21.1%)
Autism Diagnostic Interview-Revised	32 (42.1%)
Diagnosis by physician, psychologist, and speech therapist (Arkansas State Standard)	20 (26.3%)
DSM diagnosis by physician with standardized, validated questionnaires & diagnosis confirmation by the Principal Investigator	56 (73.7%)
Regression, N (%)	
Single Regression	13 (17.1%)
Multiple Regressions	32 (42.1%)
Comorbid Conditions (Parent Report), N (%)	
Neurological	50 (65.8%)
General Health	44 (57.9%)
Allergic	41 (53.9%)
Psychiatric	40 (52.6%)
Gastrointestinal	32 (42.1%)
Immune	30 (39.5%)
Growth	17 (22.4%)
Endocrine	8 (10.5%)
Cardiovascular	7 (9.2%)
Genitourinary	3 (3.9%)
Comorbid Conditions (Medical Records), N (%)	
Food Allergies/Intolerances	63 (82.9%)
Gross Motor Delay	45 (59.2%)
Chronic Constipation	42 (55.3%)
Fatigue/Exercise Intolerance	34 (44.7%)
Recurrent Infections (AAAAI Criteria)	28 (36.8%)
Epilepsy	22 (28.9%)
Hypogammaglobinemia	17 (22.4%)
Failure to Thrive	8 (10.5%)
Down Syndrome	3 (3.9%)
Tic Disorder	0 (0%)
Treatments, N (%)	
Gastrointestinal Medications	47 (61.8%)
Mineral Supplements	35 (46.1%)
Melatonin	27 (35.5%)
Allergy/Asthma Medications	24 (31.6%)
Antiepileptic Medications	19 (25.0%)
Antimicrobial Medications	17 (22.4%)
Immunomodulatory Medications	17 (22.4%)
Other Psychotropic Medications	15 (19.7%)
Hormone Supplementation	7 (9.2%)
Stimulant	7 (9.2%)
Thyroid Supplementation	7 (9.2%)
Alpha-adrenergic agonists	6 (7.9%)
Selective Serotonin Reuptake Inhibitors	4 (5.3%)
Beta Blocker	2 (2.6%)
Anticholinergic	1 (1.3%)
Dietary Formula	1 (1.3%)

Comorbid conditions were both derived from a parent reported medical questionnaire and from review of conditions diagnosed in the medical records. Regression (defined as loss of already obtained skills) was defined in detail in our questionnaire. Questions regarding regression included the timing, specific skills lost, duration of the regression, trigger and whether there were multiple regressions. Since this paper is not specifically on regression, we have not summarized these details in the table. This method for assessing medical comorbidies has been used in several of our previous studies (Frye et al. 2016b, d; Delhey et al. 2017; Frye et al. 2017b). Since there are numerous medical conditions, we summarized them into categories. Gastrointestinal disorders included constipation, diarrhea, stomach/abdominal pain, gastroesophageal reflux disease, vomiting, and feeding problems. Allergic disorders included allergies, asthma, breathing problems, other lung problems, and allergic skin conditions. Psychiatric disorders included depression, bipolar, anxiety disorder, obsessive compulsive disorder, attention deficit with or without hyperactivity. Immune disorder included ear infections, sinusitis, throat infections and other immune problems. Neurological disorders included hearing problems, headaches, vision problems, microcephaly, macrocephaly, seizures, opthalmoplegia, strabismus, cerebral palsy, tics, and muscle weakness. General health disorders included dental problems, fatigue, lack of sweating, exercise intolerance, heat intolerance, bone and joint problems, and blood problems and anemia. Cardiovascular disorders included heart conditions, syncope, congenital heart disease, and heart failure. Growth disorders included growth hormone deficiency, failure to thrive, short stature, tall stature, overweight and obesity. Endocrine disorders includes thyroid or other endocrine problems. Genitourinary disorders includes kidney, bladder, and genital problems.

Inclusion criteria were (i) age 3 to 14 years of age and (ii) ASD diagnosis. Exclusion criteria were (i) chronic treatment with medications that would detrimentally affect mitochondrial function such as antipsychotic medications; (ii) vitamin or mineral supplementation exceeding the recommended daily allowance, and (iii) prematurity.

The ASD diagnosis was defined by one of the following: (i) a gold-standard diagnostic instrument such as the Autism Diagnostic Observation Schedule and/or Autism Diagnostic Interview-Revised; (ii) the state of Arkansas diagnostic standard, defined as agreement of a physician, psychologist and speech therapist; and/or (iii) Diagnostic Statistical Manual (DSM) diagnosis by a physician along with standardized validated questionnaires and diagnosis confirmation by the Principal Investigator. We have validated that this criteria captures an accurate diagnosis of ASD in our previous studies by re-evaluating a portion of the participants with the Autism Diagnostic Interview-Revised and determining that they all were well within the diagnostic criteria for ASD (Frye et al. 2016b, d; Delhey et al. 2017; Frye et al. 2017b).

### Historical healthy controls

Historical controls of similar age and gender included 68 healthy individuals without neurological disease as described in previous studies (Goldenthal et al. 2015). Controls ranged in age from 3 to 21 years of age [mean (SD) 10.1 years (4.6 years)] with 33 (49%) being female. These controls were evaluated with the same methodology used in this study so that the data from the controls were truly comparable to the data collected in the current study. In a previous report, it was found that there was no correlation between enzyme activities and age and no difference in protein activities across ethnicity or race in both controls and mitochondrial disease patients (Goldenthal et al. 2012).

### Contemporaneous healthy controls

Contemporaneous controls of similar and age and gender included 14 generally healthy children without significant chronic health conditions. These controls ranged in age from 1 to 15 years of age [Mean (SD) 9.1 years (4.8 years)] with 6 (42%) being female. These individuals underwent the Vineland Adaptive Behavior Scale 2nd Edition (VABS) evaluation as well as an evaluation for mitochondrial function so their data could be included in the analysis examining the relationship between the VABS and variation in mitochondrial function in order to investigate whether the patterns found for children with ASD were in line with those found in typically developing individuals.

### Cognitive and behavioral outcome measures

As mentioned in our previous study (Frye et al. 2016d), our research staff was trained by a multispecialty team consisting of two licensed psychologists and a speech therapist prior to performing assessments. During the study a research psychologist supervised research staff and provided feedback and retraining if necessary.

Observer rated measures included language and the Ohio Autism Clinical Impression Scale (OACIS). Parents completed the Aberrant Behavior Checklist (ABC), Social Responsiveness Scale (SRS) the VABS Survey Interview Form and Childhood Behavioral Checklist (CBCL).

Language was assessed by an ability-appropriate instrument, the CELF-preschool-2, the CELF-4 or the Preschool Language Scale-5 (PLS-5). The CELF is a standardized, well-validated instrument that has been used in studies on ASD (Verly et al. 2014; Edgar et al. 2015). The PLS-5 is a standardized, well-validated instrument that measures subtle changes in verbal communication, particularly in pre-verbal children (Volden et al. 2011). The standardized summary score of each instrument (mean 100, standard deviation 15) was used as the outcome measure.

The OACIS is an observer-rated scale sensitive to clinically meaningful changes in ASD symptoms (Butter and Mulick 2006) which has been shown to have good inter-rater and cross-cultural reliability (Choque Olsson and Bolte 2014) and has been used in several ASD clinical trials (Frye et al. 2015). The VABS is a reliable and valid measure of the ability to perform age-appropriate everyday skills, including communication, daily living, social and motor skills, through a 20–30 min structured interview with a caretaker (Frye et al. 2013a). The ABC is a 58-item questionnaire (Frye et al. 2013a) that measures disruptive behaviors and has convergent and divergent validity (Kaat et al. 2014). The SRS is a 65-item questionnaire that measures the severity of social skill deficits across five domains (Constantino 2002) which has been shown to have good correspondence to the gold-standard instrument (Murray et al. 2011). The CBCL measures general behavioral problems in children and has been described as robust for use in measuring behavior in children with ASD (Hanratty et al. 2015).

### Measures of mitochondrial function

The buccal cells were collected using Catch-All Buccal Collection Swabs (Epicentre Biotechnologies, Madison, WI). Four swabs were collected by firmly pressing a swab against the inner cheek while twirling for 30s. Swabs were clipped and placed in 1.5 ml microcentrifuge tubes that were labeled and placed on dry ice for overnight transportation to the Goldenthal laboratory.

Buccal extracts were prepared using an ice-cold buffered solution (Buffer A, ABCAM) containing protease inhibitor cocktail and membrane solubilizing non-ionic detergent and cleared of insoluble cellular material by high speed centrifugation at 4 °C. Duplicate aliquots of the protein extract were analyzed for protein concentration using the bicinchoninic acid method (Pierce Biotechnology, Rockford, IL). Samples were typically stored at −80 °C for up to 1 week prior to enzymatic analysis.

Dipstick immunocapture assays measured ETC Complex I activity using 50 μg of extracted protein (Ezugha et al. 2010; Goldenthal et al. 2012; Yorns et al. 2012). Signals were quantified using a Hamamatsu immunochromato reader (MS 1000 Dipstick reader). Raw milliAbsorbance results were corrected for protein concentration and data were expressed as percentages of the values obtained with control extracts run on the same assay. ETC Complex IV and CS activity were assessed using standard spectrophotometric procedures in 0.5 ml reaction volume. Specific activities of respiratory complexes were initially expressed as nanomoles/min/mg protein. For comparison to controls, Complex I and IV activities were normalized to CS activity.

### Statistical analysis

Analyses were performed using SAS 9.4 (SAS Institute Inc., Cary, NC). Graphs were produced using Excel version 14.0 (Microsoft Corp, Redmond, WA). This analysis is modeled on our recently analysis of bioenergetic variation in a genetic syndrome (Frye et al. 2016a).

Normal control values were based upon the established historical controls from the Goldenthal laboratory published previously. The two-tailed 95% normative range was considered within 1.96 +/− standard deviation (SD) of the mean, meaning that abnormal values were above 97.5% or below 2.5% of the normal distribution as defined by the historical control mean and standard deviation for each ETC complex activity measured. For comparison to normal historical controls, ETC complex activity was normalized to CS activity.

Abnormalities in ETC complex activity were analyzed in several ways. First, the number of values outside of the normal range was calculated for each ETC complex measured. The binomial probability was calculated to determine if the number of values outside the normal range were significantly different than expected by chance. Second, the ASD group mean complex activity values were compared to the entire control population using t-tests. Third, a difference in the group variance was compared using F-tests.

To better understand the relationship between cognitive and behavioral indexes and mitochondrial enzyme activity we used a general linear model with the cognitive and behavioral indexes as the dependent measure and mitochondrial enzyme activity as predictor variables. ETC complex activity was not normalized in this analysis so the effect of CS could be differentiated from the effect of individual complex activity. Since there is data suggesting that mitochondrial enzyme activity both abnormal and below normal is related to ASD, a second order polynomial is used to model the predictor variables in order to account for a potential curvilinear relationship. The generalized linear model module ‘genmod’ in SAS was used with an alpha of 0.05 with a backward stepwise elimination of non-significant variables. Since adaptive behavior is known to affect social (i.e., SRS (Hus et al. 2013)) and behavior (e.g. ABC (Rattaz et al. 2015)) symptoms in ASD, the composite score of the VABS was used as a covariate for the SRS, ABC, CBCL and OASIS outcome measures.

## Results

### Mitochondrial function in autism spectrum disorder

**Fig. 1:**
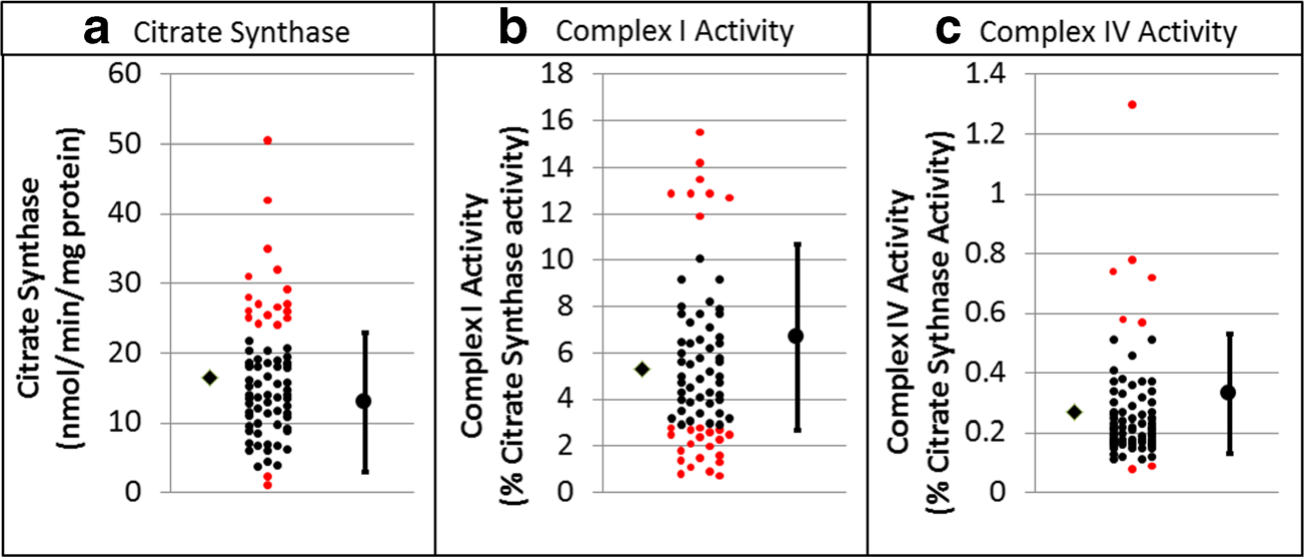
Activity of Citrate Synthase and activities of Complex I and IV normalized to Citrate Synthase for children with Autism Spectrum Disorder (ASD). For each graph the control mean and minimal and maximum limits of normal controls are provided on the *right*. Individual participant data is provided in the *center* of each graph with individuals having values outside of the normal range highlighted in *red*. The mean of the ASD group is depicted on the left of the individual data points as *diamond*

Table 2 outlines the results of the statistical analyses. Overall 62% of ASD individuals showed mitochondrial enzyme activity outside the control range (Fig. 1). Complex I activity was outside of the control range for 39% of ASD participants, which is significantly more than expected by chance alone. Variation in Complex I activity was significantly greater in ASD participants as compared to the control group and mean Complex I activity for ASD participants was significantly lower than the control group. Complex IV activity was outside the normal range for 11% of the participants, which is significantly more than expected by chance alone. Mean Complex IV activity was significantly lower in ASD participants as compared to the control group, and variation was significantly greater in the ASD group as compared to the control group. The ratio of Complex I to Complex IV was abnormal in 24% of the ASD participants. The average ratio was not significantly different than controls but the variation was greater in the ASD group as compared to the control group.

**Table 2:** Statistical analysis of enzyme activity in children with Autism Spectrum Disorders (ASD). On average, Citrate synthase was significantly higher and Complex I and IV activity normalized to citrate synthase activity were significantly lower for ASD participants as compared to controls. The variability in enzyme activity was significantly higher for all enzymes in ASD participants as compared to controls. Also significantly more enzyme activity values were outside the normal range for ASD participants as compared to the control range

	Citrate synthase (nmol/min/mg protein	Complex I activity (% citrate synthase activity)	Complex IV activity (% citrate synthase activity)	Complex I/IV ratio	Any abnormal
Control Mean (SD)	12.9 (5.1)	6.7 (2.0)	0.33 (0.10)	21.2 (9.1)	
ASD Mean (SD)	16.5 (9.0)	5.4 (3.5)	0.27 (0.19)	25.3 (21.4)	
Mean Difference (t_142_, p)	**3.04, <0.005**	**2.80, <0.01**	**2.26, <0.05**	1.51, =0.13	
Variance Difference (F_75,67_,p)	**3.14, <0.0001**	**3.12,<0.0001**	**3.52, <0.0001**	**5.53,<0.0001**	
Normal Range	2.9–22.9	2.8–10.6	0.13–0.53	3.4–39	
% Above Normal, *p* value	**22% (17/76) < 0.0001**	**11% (8/76) < 0.0001**	**8% (6/76) < 0.005**	**7% (5/76) < 0.01**	**41% (31/76) < 0.0001**
% Below Normal, *p* value	3% (2/76), 0.57	**28% (21/76) < 0.0001**	3% (2/76) 0.56	**17% (13/76) < 0.0001**	**45% (34/76) < 0.0001**
% Outside Normal, *p* value	**24% (19/76) < 0.0001**	**39% (29/76) < 0.0001**	**11% (8/76)** **=0.01**	**24% (18/76) < 0.0001**	**62% (47/76) < 0.0001**

Statistically significant values are represented in bold

Table 3 outlines the overlap of complex function abnormalities within the 47 ASD participants with atypical mitochondrial function. Overall, most, 57% (27/47), of the ASD participants with mitochondrial enzyme abnormalities had only one enzyme affected, while 32% (15/47) had two enzymes affects and 7% (3/47) had all three enzymes affected. Complex I and Citrate Synthase (CS) were isolated a majority of the time while Complex IV abnormalities were commonly associated with either a Complex I or CS abnormality. Table 4 provides the details of the overlap of the underactivity and overactivity of specific mitochondrial enzymes. Although some interesting patterns emerge, the number of samples limit conclusions that can be made from these patterns.

**Table 3:** Overlap of mitochondrial enzyme abnormalities in Autism Spectrum Disorder

Enzyme affected	Overlap with other enzymes	
Complex I (*n* = 29)	Only Complex I Affected	69% (20/29)
	Also Complex IV Affected	14% (4/29)
	Also Citrate Synthase Affected	28% (8/29)
Complex IV (*n* = 8)	Only Complex IV Affected	38% (3/8)
	Also Complex I Affected	50% (4/8)
	Also Citrate Synthase Affected	50% (4/8)
Citrate Synthase (*n* = 19)	Only Citrate Synthase Affected	53% (10/19)
	Also Complex I Affected	42% (8/19)
	Also Complex IV Affected	21% (4/19)

**Table 4:** Details of the overlap of underactivity and overactivity of mitochondrial enzymology function

Enzyme affected	Cpx I Abn	Cpx I under active	Cpx I over active	Cpx IV Abn	Cpx IV under active	Cpx IV over active	Citrate Syn Abn	Citrate Syn under active	Citrate Syn over active
Complex I (*n* = 29)		72% (21)	28% (8)	14% (4)	3% (1)	10% (3)	28% (8)	7% (2)	21% (6)
Underactive (*n* = 21)				14% (3)	5% (1)	10% (2)	29% (6)	10% (2)	19% (4)
Overactive (*n* = 8)				13% (1)	0% (0)	13% (1)	25% (2)	0% (0)	25% (2)
Complex IV (*n* = 8)	50% (4)	38% (3)	13% (1)		25% (2)	75% (6)	50% (4)	25% (2)	25% (2)
Underactive (*n* = 2)	50% (1)	50% (1)	0% (0)				100%(2)	0% (0)	100% (2)
Overactive (*n* = 6)	50% (3)	33% (2)	17% (1)				33% (2)	33% (2)	0% (0)
Citrate Syn (*n* = 19)	42% (8)	32% (6)	11% (2)	21% (4)	11% (2)	11% (2)		11% (2)	89% (17)
Underactive (*n* = 2)	100% (2)	100% (2)	0% (0)	100% (2)	0% (0)	100%(2)			
Overactive (*n* = 17)	35% (6)	24% (4)	12% (2)	12% (2)	12% (2)	0% (0)			

**Fig. 2:**
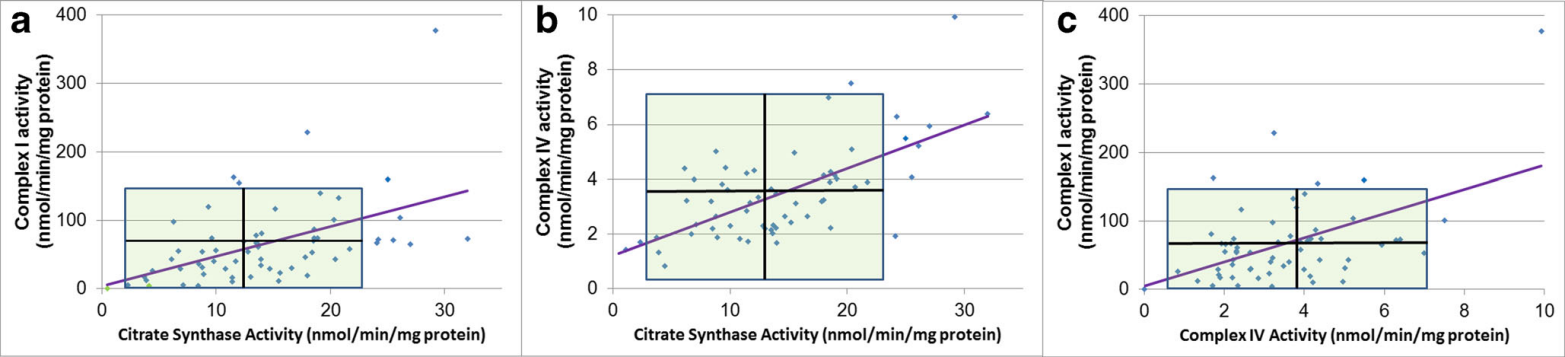
Mitochondrial enzymes activities (uncorrected) demonstrate moderate correlations. *Shaded area* represents the normal range with the *horizontal* and *vertical black lines* representing the mean of the control group

Mitochondrial enzymes demonstrated moderate correlations with one another (Fig. 2). CS activity was positively correlated with activity of Complex I (*r* = 0.47, *p* = 0.0001) and Complex IV (*r* = 0.65, *p* < 0.0001) and Complex I activity was positively correlated with activity of Complex IV (*r* = 0.50, *p* < 0.0001).

### Relationship between mitochondrial activity and symptomatology

**Fig. 3:**
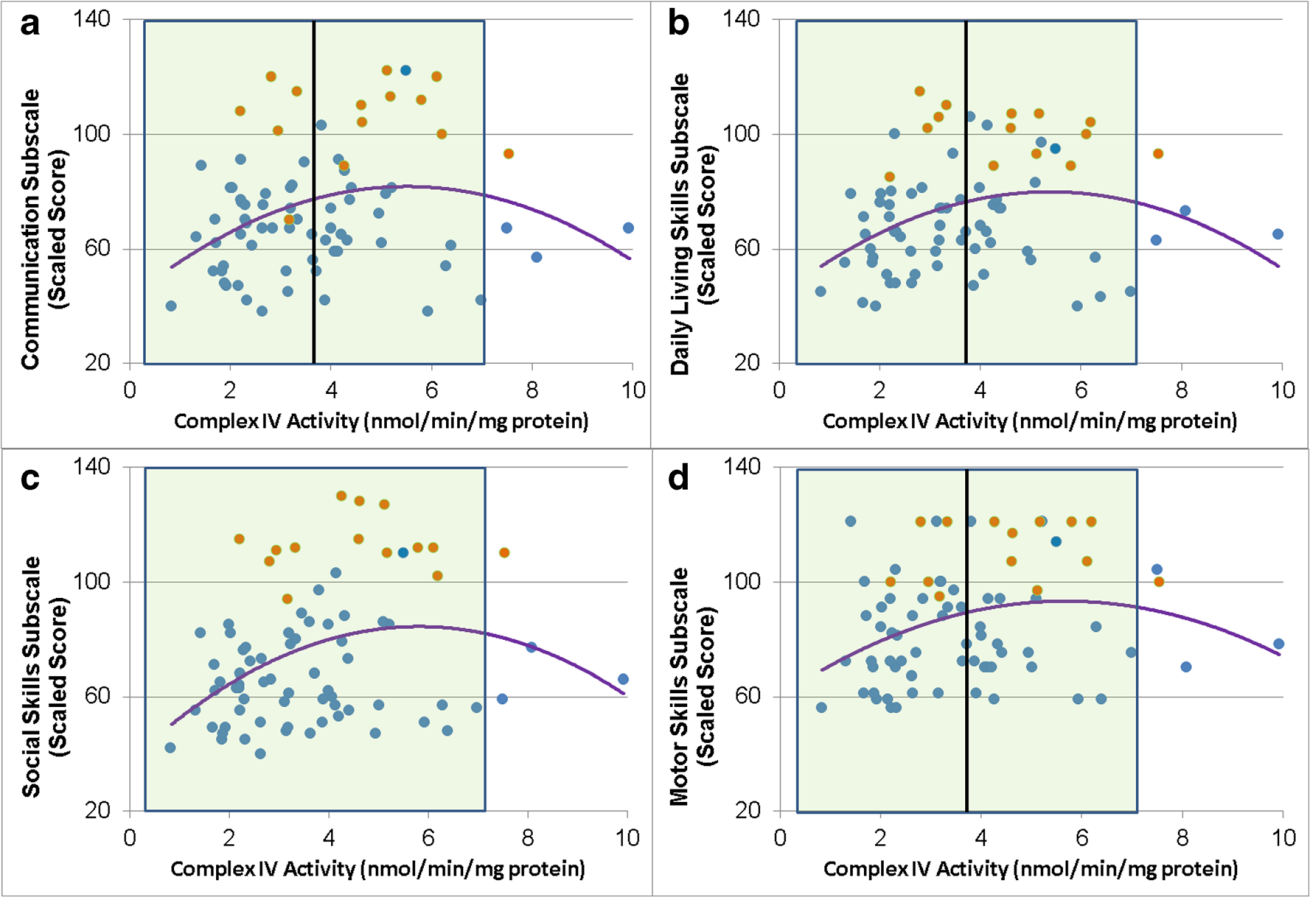
Vineland Adaptive Behavior Scale subscales are related to uncorrected Complex IV activity. *Shaded area* represents the normal range with the *vertical black line* representing the mean of the control group. *Blue points* represent children with ASD while the *orange points* represent typically developing control children

The VABS Communication, Daily Living, Social and Motor subscales and the overall Adaptive Behavior Composite were all related to Complex IV activity in a non-linear manner (Fig. 3). Communication [Linear χ^2^ (1) = 11.58, *p* < 0.001; Quadratic χ^2^ (1) = 10.58, *p* = 0.001; Fig. 3a], Daily Living [Linear χ^2^ (1) = 11.30, *p* < 0.001; Quadratic χ^2^ (1) = 10.35, *p* = 0.001; Fig. 3b], Social skills [Linear χ^2^ (1) = 12.22, *p* < 0.001; Quadratic χ^2^ (1) = 10.60, *p* = 0.001; Fig. 3c] and Motor skills [Linear χ^2^ (1) = 9.11, *p* < 0.01; Quadratic χ^2^ (1) = 6.53,*p* = 0/01; Fig. 3d] were related to Complex IV activity such either relatively higher or lower Complex IV activity was related to poorer skills. Complex IV activity also demonstrated a similar non-linear relationship with the Adaptive Behavior Composite [Linear χ^2^ (1) = 12.73, *p* < 0.0005; Quadratic χ^2^ (1) = 11.35, *p* < 0.001].

**Fig. 4:**
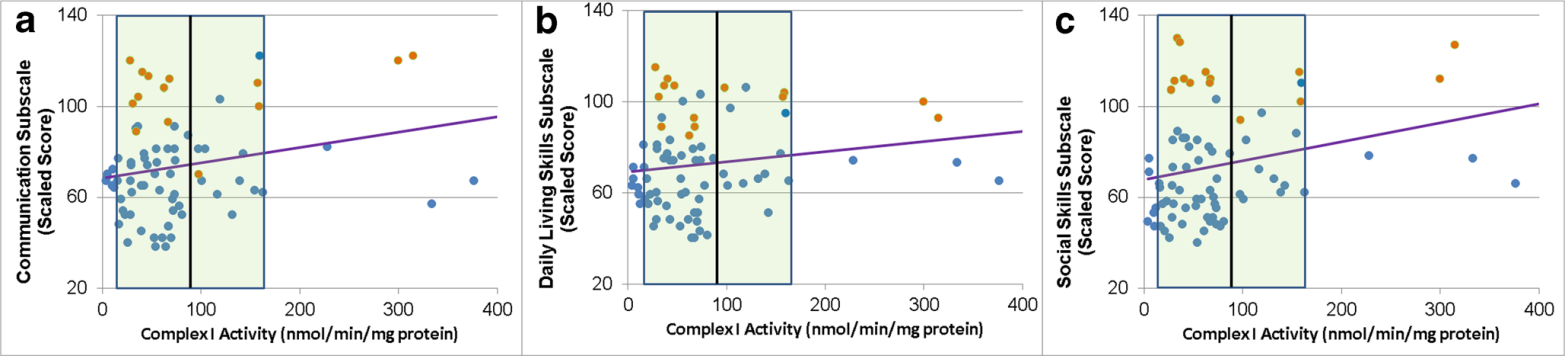
Vineland Adaptive Behavior Scale subscales are related to uncorrected Complex I activity in a linear manner. *Shaded area* represents the normal range with the *vertical black line* representing the mean of the control group. *Blue points* represent children with ASD while the *orange points* represent typically developing control children

Complex I was also concurrently related to VABS in a linear manner (Fig. 4) for the Adaptive Behavior Composite [Linear χ^2^ (1) = 5.00, *p* < 0.05] and VABS subscales except motor skills. As see in Fig. 4, better Communication [Linear χ^2^ (1) = 5.72, *p* < 0.05], Daily Living [Linear χ^2^ (1) = 3.71, *p* = 0.05], and Social skills [Linear χ^2^ (1) = 10.60, *p* = 0.001] were linearly related to higher Complex I activity.

**Fig. 5:**
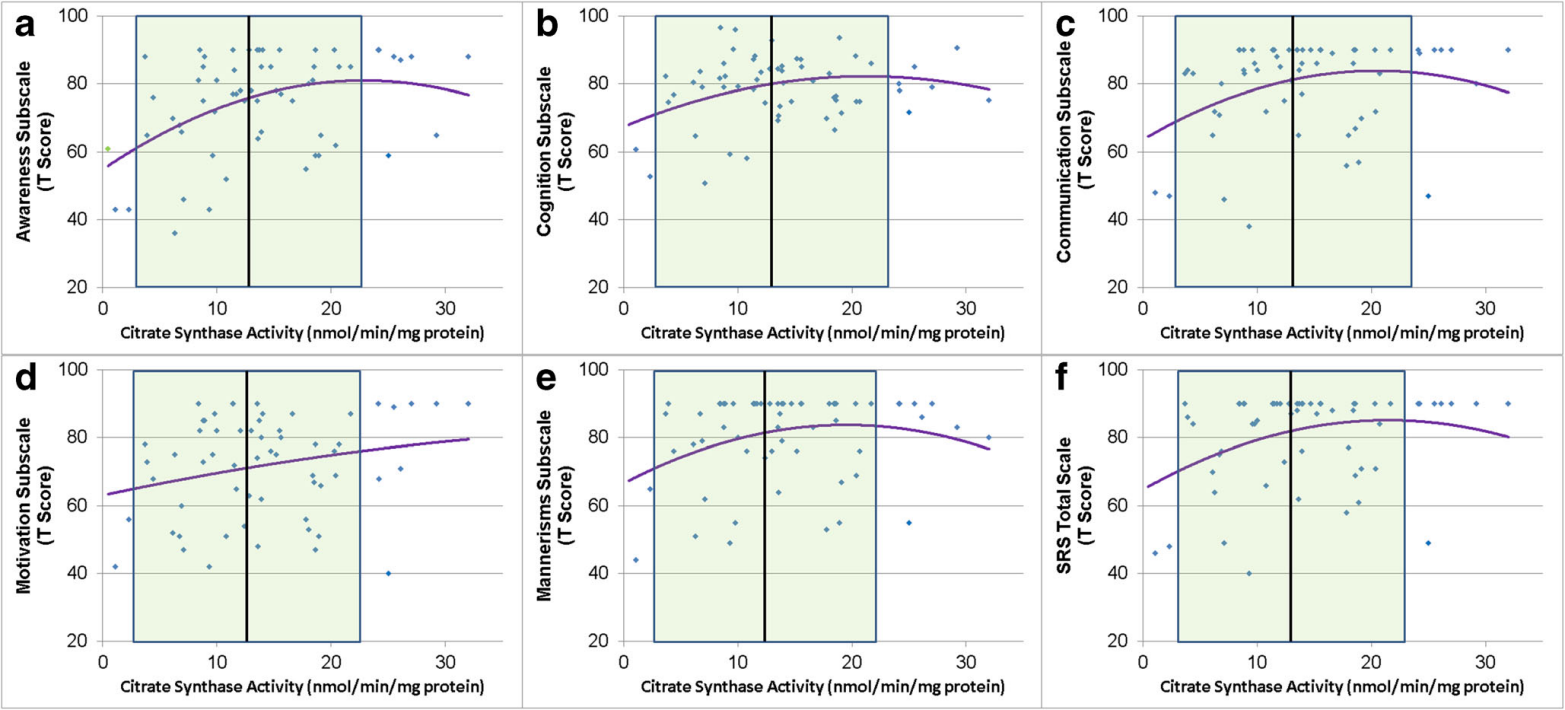
Social Responsiveness Scale subscales are related to Citrate Synthase activity. *Shaded area* represents the normal range with the *vertical black line* representing the mean of the control group

SRS total and subscales were related to CS activity in a non-linear manner such that relatively worse social function was associated with higher CS activity (Fig. 5) when level of adaptive function was taken into account [Awareness Linear χ^2^(1) = 10.60, *p* = 0.001, Quadratic χ^2^(1) = 6.97, *p* < 0.01, Adaptive Behavior χ^2^(1) = 18.84, *p* < 0.0001; Cognition Linear χ^2^(1) = 4.15, *p* < 0.05, Quadratic χ^2^(1) = 6.02, *p* = 0.01, Adaptive Behavior χ^2^(1) = 32.28, *p* < 0.0001; Communication Linear χ^2^(1) = 6.21, *p* = 0.01, Quadratic χ^2^(1) = 6.10, *p* = 0.01, Adaptive Behavior χ^2^(1) = 30.75, *p* < 0.0001; Motivation Linear χ^2^(1) = 4.75, *p* < 0.05, Quadratic χ^2^(1) = 3.59, *p* = 0.05, Adaptive Behavior χ^2^(1) = 20.14, *p* < 0.0001; Mannerisms Linear χ^2^(1) = 7.41, *p* < 0.01, Quadratic χ^2^(1) = 12.18, *p* = 0.0005, Adaptive Behavior χ^2^(1) = 25.91, *p* < 0.0001; Total Linear χ^2^(1) = 8.74, *p* < 0.005, Quadratic χ^2^(1) = 10.27, *p* = 0.001, Adaptive Behavior χ^2^(1) = 34.93, *p* < 0.0001].

Mitochondrial enzyme activity was not related to the other behavioral or language assessments.

## Discussion

Previous studies have suggested that abnormal mitochondrial function is associated with ASD but the exact nature of this mitochondrial dysfunction and how it is associated with ASD symptoms is not clear. In this study we have measured mitochondrial enzyme activity on the one of the largest cohorts of individuals with ASD studied to date with concurrent measurement of symptoms in a subset. This study demonstrates that there is an expanded variation in mitochondrial enzyme activity in children with ASD, with enzyme activity that is both higher and lower than found in typically developing individuals. These variations are associated with ASD symptoms as well as measures of general development with some of these relationships non-linear such that both relatively higher and lower enzyme activity values are similarly associated with unfavorable alternations in behavior and development. A strength of this study is that the children in this study were not taking any medications or supplements that could potentially influence mitochondrial activity. Indeed, many medications commonly used to treat children with ASD, such as antipsychotics, can inhibit mitochondrial function (Wallace 2014) and many supplements commonly used in ASD can alter mitochondrial enzyme activity (Delhey et al. 2017).

In classic mitochondrial disease, the mitochondrion is thought to be severely dysfunctional with significant depression in mitochondrial respiration. Criteria such as the modified Walker’s criterion reflects this notion and are commonly used to diagnose mitochondrial disease, although other criteria like the Morava criteria, which are more clinically based, are also sometimes used (Frye and Rossignol 2011). Classic mitochondrial disease is often found to have a genetic etiology, although this seems to be more relevant to adults with classic forms of mitochondrial disease than children. It is also being recognized that many genetic and non-genetic disorders can cause mitochondrial dysfunction that can may require treatments targeting the mitochondria (Niyazov et al. 2016). Additionally, the great majority of children with ASD who are diagnosed with mitochondrial disease do not have a simple genetic alteration to explain their problem in mitochondrial function (Rossignol and Frye 2012). Many of the children with ASD in this study were found to have a decrease in mitochondrial activity, particularly below normal activity in Complex I. This is consistent with muscle biopsies of children with ASD diagnosed with mitochondrial disease, where the most common ETC deficit was Complex I (Rossignol and Frye 2012).

Possibly unique to ASD is mitochondrial enzyme overactivity. Increases in ETC complex activity, rather than deficiencies in ETC complex activity, has been reported in several tissues derived from individuals with ASD, including muscle (Frye and Naviaux 2011; Frye 2012a, b), skin (Frye et al. 2013b), buccal (Legido et al. 2013; Goldenthal et al. 2015; Frye et al. 2016a) and brain (Palmieri et al. 2010) tissue. This increase in respiratory function is consistent with our cell line model of mitochondrial function in ASD. Indeed, we have repeatedly demonstrated in the laboratory that a subset of lymphoblastoid cell lines derived from children with ASD have respiratory rates approximately twice that of control cell lines (Rose et al. 2014a, b, 2015a, b; Frye et al. 2016c, 2017a; Rose et al. 2017). We recently demonstrated that this alteration in respiration is associated with worse repetitive behaviors (Rose et al. 2017). We believe that this increase in respiratory rate may be an adaptation designed to resistant toxicants associated with ASD, perhaps because of previous exposure to environmental toxicants (Rose et al. 2014a, b, 2017; Frye et al.  2017a). We have also demonstrated that this increased respiratory rate results in an increased susceptibility of the mitochondrial to in vitro increases in ROS (Rose et al. 2014a, b). This study confirms the association with increased ETC complex activity and ASD. Further research will be needed to better understand this unique alteration in mitochondrial physiology.

We found a greater than expected number of patients with underactive and/or overactive mitochondrial activity. This suggests that involvement of the mitochondria in psychiatric and neurodevelopmental disease may be different than what is commonly defined as mitochondrial disease, suggesting that the term mitochondrial dysfunction may be more appropriate to describe the expanded spectrum of bioenergetic variation associated with some disease, particular psychiatric and neurodevelopment disorders. In psychiatric and neurodevelopmental disease it may be that mitochondrial respiration is modulated by a variety of factors, most of which are not clear at this time. However, there are several examples of molecular pathways that are dysfunctional in psychiatric, neurologic and neurodevelopmental disease that modulate mitochondrial function, including mTOR (Zheng et al. 2016), PTEN (Missiroli et al. 2016), PINK1 (Gomez-Sanchez et al. 2016) and DISC1 (Norkett et al. 2016). This data provides new insight into the manner in which mitochondrial activity may be associated with ASD symptoms.

Although the modulation of mitochondrial activity could be secondary to abnormalities in other molecular pathways, increased or decreased mitochondrial respiration could certainly adversely affect cellular function by either resulting in insufficient energy production and/or increased reactive oxygen species (ROS) production that could damage cellular enzymes, membranes and/or genetic material. Since neurons have very high energy demands and are vulnerable to ROS, the brain may be particularly vulnerable to modulation of mitochondrial function, potentially resulting in changes in behavior and development, such as symptoms associated with ASD. Atypical variation in mitochondrial function could explain why at least some children with ASD respond to treatments for mitochondrial disease and oxidative stress (Frye and Rossignol 2014) and why some children with ASD have multisystem disease, including the disruption of function in high energy tissues such as the gastrointestinal and immune systems (Frye and Rossignol 2016).

Mitochondrial disorders can make an individual more susceptible, immunologically and neurologically, to toxins, toxicants, pathogens, and other insults (Rossignol and Frye 2012; Legido et al. 2013). Dysregulation of the mitochondrial respiratory mechanism (i.e., mitochondrial dysfunction) has been described in patients with neurodegeneration, oxidative stress and neuroinflammation (Manucha 2017). When mitochondrial dysfunction results in a critical shortage of energy, it can cause an increase in ROS, free radicals, lactic acid, cerebral edema, and inflammation, which, in turn, can drive microglial activation and subsequent cell loss (Vijitruth et al. 2006). Examples of these interactions between mitochondrial dysfunction, oxidative stress and inflammation have been demonstrated in children with ASD in several studies (Rossignol and Frye 2014) including in postmortem brain tissue (Rose et al. 2012). This framework would indeed be consistent with studies which suggestion that the etiology of ASD involves genetic susceptibility combined with environmental triggers (Hallmayer et al. 2011).

The above speculations must be tempered with the limitations of this study. We have only demonstrated an association between behavior and biomarkers of mitochondrial activity. This association does not imply causation. Indeed, there may be many other factors that influence both mitochondrial activity and behavior. In addition, both behavior and mitochondrial function demonstrate significant variability. This indeed suggests that further research will be needed to determine more details of this relationship. For example, there may be several subgroups of children with specific medical conditions and ASD which have abnormal mitochondrial function such as those with epilepsy or Downs syndrome. Despite these limitations, this report helps defined a connection between behavior and biology which is very intriguing. Further studies, using larger samples sizes will be needed.
